# Leaf photosynthetic pigment as a predictor of leaf maximum carboxylation rate in a farmland ecosystem

**DOI:** 10.3389/fpls.2023.1225295

**Published:** 2023-07-04

**Authors:** Yue Li, Qingtao Wang, Taimiao Fu, Yunfeng Qiao, Lihua Hao, Tao Qi

**Affiliations:** ^1^School of Earth Science and Engineering, Hebei University of Engineering, Handan, China; ^2^School of Landscape and Ecological Engineering, Hebei University of Engineering, Handan, China; ^3^Institute of Geographic Sciences and Natural Resources Research, Chinese Academy of Sciences, Beijing, China; ^4^School of Water Conservancy and Hydropower, Hebei University of Engineering, Handan, China

**Keywords:** leaf chlorophyll content, leaf carotenoid content, leaf nitrogen content, maximum rate of carboxylation, photosynthetic capacity

## Abstract

The leaf maximum rate of carboxylation (V_cmax_) is a key parameter of plant photosynthetic capacity. The accurate estimation of V_cmax_ is crucial for correctly predicting the carbon flux in the terrestrial carbon cycle. V_cmax_ is correlated with plant traits including leaf nitrogen (N_area_) and leaf photosynthetic pigments. Proxies for leaf chlorophyll (Chl_area_) and carotenoid contents (Car_area_) need to be explored in different ecosystems. In this study, we evaluated the relationship between leaf maximum rate of carboxylation (scaled to 25°C; V_cmax25_) and both leaf N_area_ and photosynthetic pigments (Chl_area_ and Car_area_) in winter wheat in a farmland ecosystem. Our results showed that V_cmax25_ followed the same trends as leaf Chl_area_. However, leaf N_area_ showed smaller dynamic changes before the flowering stage, and there were smaller seasonal variations in leaf Car_area_. The correlation between leaf V_cmax25_ and leaf Chl_area_ was the strongest, followed by leaf Car_area_ and leaf N_area_ (R^2^ = 0.69, R^2^ = 0.47 and R^2 ^= 0.36, respectively). The random forest regression analysis also showed that leaf Chl_area_ and leaf Car_area_ were more important than leaf N_area_ for V_cmax25_. The correlation between leaf V_cmax25_ and N_area_ can be weaker since nitrogen allocation is dynamic. The estimation accuracy of the V_cmax25_ model based on N_area_, Chl_area_, and Car_area_ (R^2 ^= 0.75) was only 0.05 higher than that of the V_cmax25_ model based on Chl_area_ and Car_area_ (R^2 ^= 0.70). However, the estimation accuracy of the V_cmax25_ model based on Chl_area_ and Car_area_ (R^2 ^= 0.70) was 0.34 higher than that of the V_cmax25_ model based on N_area_ (R^2 ^= 0.36). These results highlight that leaf photosynthetic pigments can be a predictor for estimating V_cmax25_, expanding a new way to estimate spatially continuous V_cmax25_ on a regional scale, and to improve model simulation accuracy.

## 1 Introduction

Farmland ecosystems play an important role in the carbon cycle of terrestrial ecosystems (Robertson et al., 2000). However, the carbon flux of farmland ecosystems is one of the main uncertainties in global terrestrial carbon cycle research and is significantly affected by human activities (Lal, 2001; Bondeau et al., 2007; Taylor et al., 2013). High-quality simulations of the carbon budget of farmland ecosystems are beneficial for future projections of climate change and crop yield (Houborg et al., 2015; Bonan and Doney, 2018). Process-based terrestrial biosphere models (TBMs) are effective tools for estimating changes in the ecosystem carbon budget. However, currently there is still significant uncertainty in simulating the impact of climate change on terrestrial carbon flux (Smith and Dukes, 2012; Anav et al., 2015; Li et al., 2018a). Approximately 90% of carbon and water fluxes in biosphere and atmospheric occur through photosynthesis, and the photosynthetic module is an important part of TBMs (Zhu et al., 2016). Photosynthetic rate is a primary source of uncertainty in terrestrial carbon dynamic modelling because of the lack of in-depth research on photosynthesis and field observation data (Dietze, 2014).

To simulate photosynthetic rate, most TBMs used a kinetic enzyme model based on Farquhar–von Caemmerer–Berry (FvCB) (Farquhar et al., 1980; Jin et al., 2023). The maximum rate of carboxylation (V_cmax_) and the maximum rate of electron transport (J_max_) are two key photosynthetic parameters in FvCB model. V_cmax_ represents the maximum rate of Ribulose‐1,5‐Bisphosphate (RuBP) carboxylation catalyzed by Rubisco (ribulose 1,5-bisphosphate carboxylase/oxygenase) enzyme (Quebbeman and Ramirez, 2016). J_max_ is the rate of RuBP regeneration through the electron transport chain (Voncaemmerer and Farquhar, 1981; Sharkey et al., 2007). In process-based models, V_cmax_ plays a critical role in constraining photosynthetic rates (Lebauer et al., 2013). Previously, V_cmax_ was assumed to be a fixed value (at the temperature of 25°C; V_cmax25_), which varied with plant functional type (PFT) in process-based models (Houborg et al., 2013; Zhang et al., 2014). Nonetheless, there are seasonal variations for V_cmax25_ (Grassi et al., 2005; Medvigy et al., 2013; Alton, 2017; Croft et al., 2017). Even for the same PFT, the difference between species is great (Dillen et al., 2012; Croft et al., 2017). Previous studies have typically used leaf nitrogen content (N) to model the photosynthetic capacity to incorporate spatiotemporal changes in V_cmax25_ (Kattge et al., 2009; Walker et al., 2014). However, it is not possible to accurately retrieve leaf nitrogen content based on remote sensing data (Knyazikhin et al., 2013). Additionally, a relationship between leaf N and V_cmax25_ cannot be applied at large scales or to different PFTs because Rubisco-N, rather than total leaf nitrogen (photosynthetic and nonphotosynthetic nitrogen pools), is more related to V_cmax25_ (Croft et al., 2017; Onoda et al., 2017; Effah et al., 2023). Nonphotosynthetic N pools can complicate the relationships between leaf V_cmax25_ and leaf N.

In recent years, leaf chlorophyll content has been retrieved relatively accurately via remote sensing (Croft et al., 2013), which plays a crucial role in capturing light energy to drive photosynthetic reactions (Yang et al., 2014; Croft et al., 2020; Huang et al., 2023). Leaf chlorophyll can effectively eliminate the influence of nonphotosynthetic N, which refers to changes in the photosynthetic active N pool (Croft et al., 2017). Leaf chlorophyll contents have been adopted to represent photosynthetic capacity in some studies (Houborg et al., 2015; Croft et al., 2017). In farmland ecosystems, Houborg et al. (2013) adopted an intermediate variable (leaf N) to demonstrate a semi- empirical relationship between the leaf V_cmax25_ and chlorophyll content. In temperate deciduous forests, Croft et al. (2017) found a direct correlation between leafV_cmax25_ and leaf Chl_area_. Luo et al. (2018) incorporated Chl_leaf_ into terrestrial biosphere models to constrain V_cmax25_ based on the relationship between V_cmax25_ and Chl_leaf_ from the work of Croft et al. (2017), and improved the temporal correlations between the measured and the estimated fluxes in a temperate deciduous forest. Strong correlations between the field-measured leaf chlorophyll content and V_cmax25_ have been reported in various PFTs (Qian et al., 2019; Lu et al., 2020; Wang et al., 2020; Qian et al., 2021; Lu et al., 2022; Liu et al., 2023b). Recent studies have found that leaf carotenoid, another major photosynthetic pigment, can improve the estimation precision for V_cmax25_ based on leaf Chl_area_. Leaf carotenoid content increases the capability of phenological monitoring, particularly in areas where seasonal variations in leaf chlorophyll content are not obvious (Wong et al., 2019). The functional relationship between the photosynthetic pigments (chlorophyll and carotenoid) and V_cmax25_ plays an important role in regional model simulations (Croft and Chen, 2018; Luo et al., 2018; Luo et al., 2019). Therefore, leaf chlorophyll and carotenoid contents should be incorporated into the V_cmax25_ model to improve the accuracy of TBMs in simulating C dynamics (Luo et al., 2019). However, the relationships between leaf pigment content (especially leaf carotenoid content) and V_cmax25_are still unclear. A large-scale spatial mapping of V_cmax25_ requires understanding how these relationships change in different PFTs.

In this study, we estimated the relationships between V_cmax25_ with leaf nitrogen and leaf pigments (chlorophyll and carotenoid) in a farmland ecosystem. Photosynthesis response curves, leaf nitrogen and leaf pigment content (chlorophyll and carotenoid contents) were observed at Yucheng (YC) Ecological Station during the 2021. We also investigated the correlations between V_cmax25_ with leaf nitrogen, chlorophyll and carotenoid contents in a farmland ecosystem. We also analyzed the relationships among these driving variables associated with V_cmax25_ and assessed their relative importance.

## 2 Materials and methods

### 2.1 Field site description

We carried out field experiments in Yucheng, Shandong Province, China (36°57′ N,116°38′ E). The station is a wheat producing area in China, which located in a warm temperate zone. The annual average temperature and precipitation are 13.1°C and 610 mm, respectively. The average temperature in January is -3°C, and the average temperature in July is 26.9°C. Precipitation occurs mainly from June to August, accounting for 69.1% of the total annual precipitation and shows a pattern of spring drought and summer floods (Zhu et al., 2020). The tidal soil is the main soil type in this area. The PH value is 8.0 and the soil organic matter content is 15.0 g kg^-1^. Mass fraction of soil total nitrogen is 0.64 g kg^-1^ (Hga et al., 2020).

### 2.2 Measurements of CO_2_ response curve

Leaf gas exchange in winter wheat was measured in a 10 × 10m subset area within a larger field. We conducted winter wheat observation experiment from day of the year (DOY) 92 (April 2) to 147 (May 27) in 2021. Leaf samples were randomly selected approximately once every seven days (**Table 1**). Three to four winter wheat leaf samples were collected weekly during the 2021 growing season. The CO_2_ response curves for leaves in winter wheat were measured by a portable gas‐exchange system (Li 6400; Li‐Cor, Inc., Lincoln, NE, USA).

**Table 1:** Leaf measurements stages and sample sizes of winter wheat at YC site in 2021.

Measurement DOY	Sample sizes	Growing stages
92 (April 2)	4	Elongation stage (≤95)
96 (April 6)	4	Booting stage (≤117)
105 (April 15)	3	Booting stage (≤117)
119 (April 29)	3	Flowering stage (≤131)
126 (May 6)	3	Flowering stage (≤131)
133 (May 13)	4	Filling stage (≤161)
140 (May 20)	4	Filling stage (≤161)
147 (May 27)	4	Filling stage (≤161)

CO_2_ response curves were observed under saturated light conditions. It took about 40 minutes to observe CO_2_ response curves. Adjust the photosynthetic photon flux density (PPFD) to 1,500 μmol m^−2^ s^−1^ (saturated light). The flow rate was maintained at 500 mmol s^-1^, and the relative humidity was set in the range of 40–80% during the measurement period. The air CO_2_ concentrations (C_a_) gradients are 380, 300, 200, 100, 50, 380, 600, 800, 1,000, and 1,200 μmol CO_2_ mol^−1^ air. Leaf samples were acclimated in a 2 × 3 cm^2^ leaf cuvette for 20 min at a temperature of 25°C and a CO_2_ concentration of 380 μmol CO_2_ mol^−1^ before measuring CO_2_ response curves. V_cmax_ and J_max_ values were estimated by an Excel tool (www.landflux.org/Tools.php) (Ethier and Livingston, 2004). Arrhenius equation (Equation 1 and **Table 2**) was used in our study to normalize V_cmax_ and J_max_ to V_cmax25_ and J_max25_ (Sharkey et al., 2007; Sharkey, 2016). The net photosynthetic rate (A_sat_) was recorded at a PPFD of 1,500 μmol m^−2^ s^−1^ and a CO_2_ concentration of 380 µmol mol^-1^.

**Table 2:** Parameters values referring to the temperature responses of leaf photosynthetic capacity.

Parameter	Value at 25°C	c	Δ H_a_
V_cmax_	1	26.355	65.33
J	1	17.710	43.90

1 indicates the value of f(T_k_)/k_25_ at 25°C.


(1) $$\text{f}\left({\text{T}}_{\text{k}}\right){\text{=k}}_{25}\text{exp(c-}\Delta {\text{H}}_{\text{a}}{\text{/RT}}_{\text{k}})$$

where k_25_ and f(T_k_) were, respectively, the values at 25°C and leaf surface temperature. c was a scaling constant (**Table 2**). ΔH_a_ referred to the activation energy. R was the molar gas constant (0.008314 kJ mol^-1^ K^-1^). T_k_ represented the absolute leaf temperature.

### 2.3 Leaf biochemistry measurements

We conducted leaf biochemical analyses (leaf nitrogen content, N_area_; leaf chlorophyll content, Chl_area;_ and leaf carotenoid content, Car_area_) on the same day as leaf A/Ci curves observations. The leaves of winter wheat were sampled from the same locations as for the A/Ci curves observations. For leaf photosynthetic pigment (chlorophyll and carotenoid) and nitrogen analyses, leaf samples were immediately packed in paper bags and were sent to chemistry laboratory. Fresh leaf weight was also recorded in chemistry laboratory. The leaf photosynthetic pigments were extracted using 95% ethanol. A Shimadzu UV-2600 spectrophotometer was used to calculate both leaf chlorophyll and carotenoid contents by measuring the absorbance at 665, 649, and 470 nm (Fargasova and Molnarova, 2010). We used the same leaves as those measured to determine the leaf photosynthetic pigments to calculate leaf nitrogen content. Dry the leaf samples at 80°C for 48 hours until a constant weight. Specific leaf area (SLA) was determined by leaf dry weights and leaf area. We ground the dried leaves into powder by a mixer mill (MM400, RETSCH, Germany). A Vario MAX CN elemental analyzer (Elementar Analyzer system, Hanau, Germany) was used to record leaf nitrogen content.

Fractions of leaf N allocated to photosynthetic components, i.e., active Rubisco (P_R_), bioenergetics pools (P_B_) and light-harvesting components (P_L_), were determined based on V_cmax_, J_max_ and leaf chlorophyll content, according to the equations reported by Niinemets and Tenhunen (1997).


(2) $${\text{P}}_{\text{R}}=\frac{{\text{V}}_{\text{cmax}}}{6.25\times {\text{V}}_{\text{cr}}\times {\text{M}}_{\text{A}}\times {\text{N}}_{\text{mass}}}$$

(3) $${\text{P}}_{\text{B}}=\frac{{\text{J}}_{\text{max}}}{8.06\times {\text{J}}_{\text{mc}}\times {\text{M}}_{\text{A}}\times {\text{N}}_{\text{mass}}}$$

(4) $${\text{P}}_{\text{L}}=\frac{{\text{C}}_{\text{C}}}{{\text{C}}_{\text{B}}\times {\text{N}}_{\text{mass}}}$$

where M_A_ referred to dry leaf mass per unit area (g m^-2^). C_c_ was leaf chlorophyll content (mmol g^-1^). N_mass_ represented nitrogen content per dry leaf mass (g g^-1^). The C_B_ value was 2.15 mmol g^-1^. The values of V_cr_ and J_mc_ were 20.5 µmol CO_2_ (g Rubisco)^-1^ s^-1^ and 156 µmol electrons (µmol cyt f)^-1^ s^-1^ at the temperature of 25°C, respectively (Niinemets et al., 1998).

### 2.4 Data analysis

In the correlation analyses, we used Pearson’s correlation coefficients to demonstrate the linear correlation strength between two variables. Pearson correlation coefficient, also known as the Pearson product-moment correlation coefficient, is represented by R in this paper. The following function was used to calculate R:


(5) $$\text{R=}\frac{\sum_{i=1}^{n}\left(x-\bar{x}\right)\left(y-\bar{y}\right)}{\sqrt{\sum_{i=1}^{n}{\left(x-\bar{x}\right)}^{2}{\left(y-\bar{y}\right)}^{2}}}$$

where n is the sample size, and R is between -1 and +1. The larger the absolute value of R, the stronger the correlation. There may be a positive (R>0) or negative (R<0) correlation between two variables.

The relationships between leaf nitrogen content and leaf photosynthetic pigments (chlorophyll and carotenoid contents) were evaluated by simple linear regressions. We used the statistical package in Origin Pro 9.0 to conduct simple linear regressions. Analysis of variance (ANOVA) was adopted to evaluate the significance of the regression equations. The statistical significance of tests was set at 0.05. The prediction variables, leading to changes in V_cmax25_, included leaf nitrogen, chlorophyll, carotenoid, and SLA. We adopted random forest regression analysis (Breiman, 2001) to discern the amount of changes in V_cmax25_. The relative importance of each predictor was evaluated by random forest regression analysis (Delgado-Baquerizo et al., 2017), which can resolve the multicollinearity problems between prediction variables. The percentage increase in the mean square error (%IncMSE) indicates the influence of replacing a predictor with a random variable on the predicted outcome, which represents the effect of predictors on the dependent variable. the original variable was more important when the random variable changed the original variance significantly.Therefore, the higher the %IncMSEof predictor, the more importance it is. Random forest package (randomForest) in R was used in our study to perform the random forest regression (http://www.r-project.org/). Multiple linear regression models were constructed to explore the effects of leaf nitrogen and photosynthetic pigments (chlorophyll and carotenoid) on variations in V_cmax25_. The performances of the V_cmax25_ models were estimated using the coefficient of determination (R^2^) between different leaf trait variables. We used SPSS^®^ version 17.0 (SPSS Inc. Chicago, IL, USA) to perform multiple linear regression analysis in our study.

## 3 Results

### 3.1 Seasonal variations in leaf photosynthetic parameters and biochemical parameters

**Figure 1:**
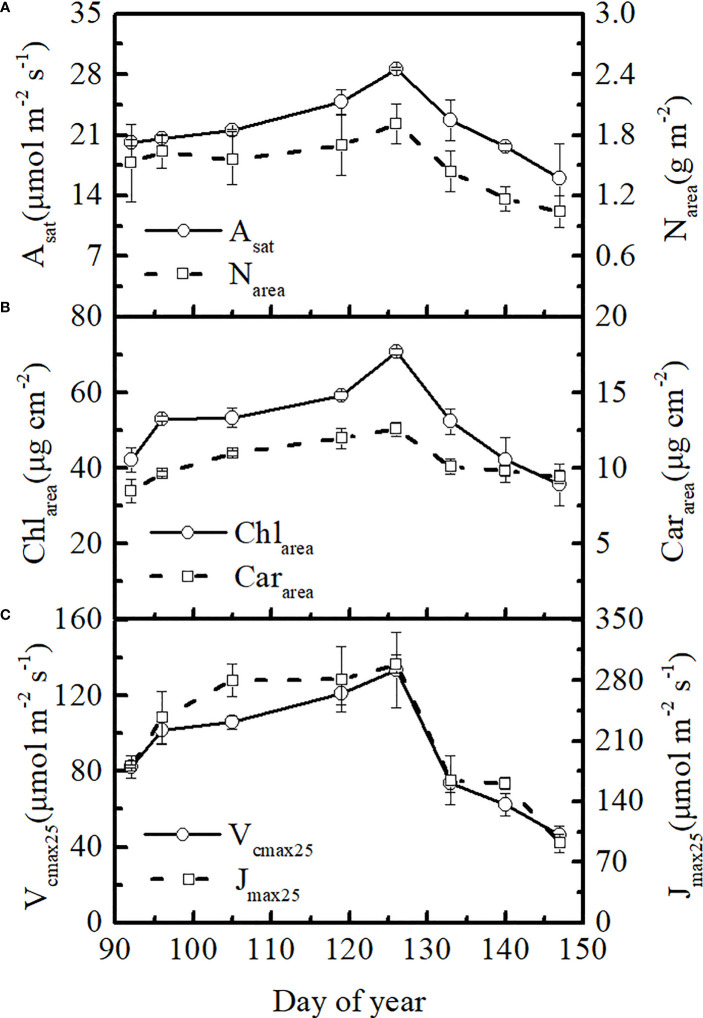
Seasonal changes in **(A)** photosynthetic rate and nitrogen content, **(B)** leaf chlorophyll and leaf carotenoid contents, and **(C)** V_cmax25_ and J_max25_ for winter wheat in 2021.

Winter wheat showed large temporal variations in leaf photosynthetic rate and V_cmax25_ in 2021. At the elongation and booting stages, the leaf A_sat_ and V_cmax25_ increased gradually before the flowering stage (on average, 42% and 62% higher on DOY 126 than on DOY 92, respectively), reaching a peak of 28.63 μmol m^-2^ s^-1^ and 133.46 μmol m^-2^ s^-1^, respectively, at the flowering stage. A_sat_ and V_cmax25_ then declined rapidly during the filling stage (on average, 79% and 190% lower on DOY 147 than on DOY 126, respectively) (**Figures 1A, C**). Temporal variations in leaf J_max25_ and V_cmax25_ were consistent (**Figure 1C**). Leaf chlorophyll content had a similar temporal variation to V_cmax25_, which gradually reached its peak at the flowering stage (on average, 68% higher on DOY 126 than on DOY 92) and rapidly declined at the filling stage (on average, 99% lower on DOY 147 than on DOY 126) (**Figure 1B**). Leaf A_sat_ and photosynthetic parameters appeared to follow the trends of leaf chlorophyll content. However, there were some differences in the seasonal patterns of leaf chlorophyll, nitrogen, and carotenoid contents. Leaf nitrogen content showed smaller dynamic changes before the flowering stage than leaf Chl_area_ (on average, 26% higher on DOY 126 than on DOY 92) and then declined rapidly at the late stage (on average, 83% lower on DOY 147 than on DOY 126) (**Figure 1A**). The peak value of leaf Car_area_ was, on average, 48% higher than that of Car_area_ on DOY 92. There were minor changes in leaf Car_area_ after the flowering stage (on average, 33% lower on DOY 147 than on DOY 126) (**Figure 1B**). Therefore, smaller seasonal changes were showed in leaf Car_area_ compared to leaf Chl_area_ in winter wheat, particularly after the flowering stage.

### 3.2 Correlation of leaf photosynthetic parameters and leaf traits variables

**Figure 2:**
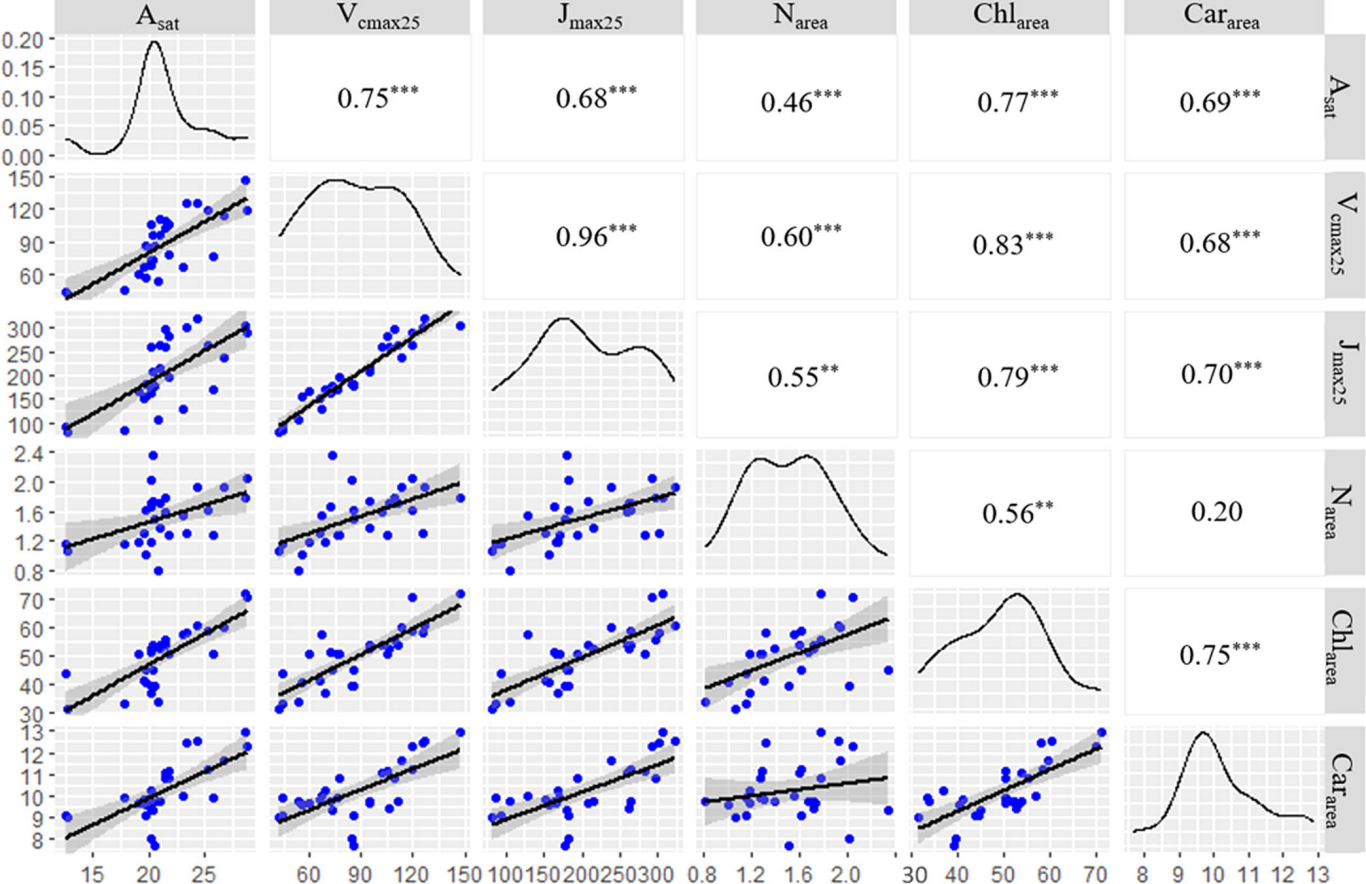
Correlation between both leaf photosynthetic rate and leaf photosynthetic capacity with different leaf traits variables. ***, ** and * represent p< 0.001, p< 0.01and p< 0.05, respectively. The caption describes all significant situations of correlation between parameters, including significant correlation (*) and extremely significant correlation (** and ***). The results in Figure 2 showed that the parameters were highly correlated (** and ***) or uncorrelated.

There were positive correlations between the leaf photosynthetic parameters (A_sat_, V_cmax25,_ and J_max25_) and leaf trait variables (N_area_, Chl_area_, and Car_area_) (**Figure 2**). The correlation coefficient between leaf V_cmax25_ and leaf Chl_area_ was the highest (0.83), followed by leaf Car_area_ (0.68) and leaf N_area_ (0.60), all of which showed significant linear positive correlations (p<0.001) (**Figure 2**). The correlations between Leaf J_max25_ were also significantly correlated with leaf traits variables (N_area_, Chl_area_, Car_area_), with correlation coefficients of 0.55 (p<0.01), 0.79 (p<0.001), and 0.70 (p<0.001), respectively. Correlations were also observed between the three leaf trait variables (**Figure 2**).

Simple linear regressions were conducted between leaf photosynthetic capacity with leaf nitrogen content, and leaf photosynthetic pigments (**Table 3**). The resultsindicated that leaf Chl_area_ accounted for 69% and 63% of the temporal variation in V_cmax25_ and J_max25_, respectively (p<0.001). Leaf Car_area_ accounted for 47% and 48% of the temporal variation in V_cmax25_ and J_max25_, respectively (p<0.001). However, there was a weak relationship between leaf N_area_ and leaf photosynthetic capacity. Leaf N_area_ accounted for only 36% and 30% of the temporal variation in V_cmax25_ and J_max25_, respectively (p<0.001) (**Table 3**). There were certain limitations to estimating V_cmax25_ based on leaf N_area_.

**Table 3:** Coefficients of determination for simple linear regressions between photosynthetic parameters with both leaf nitrogen and leaf photosynthetic pigment during 2021.

	N_area_		Chl_area_		Car_area_	
	R^2^	p	R^2^	p	R^2^	p
V_cmax25_	0.36	***	0.69	***	0.47	***
J_max25_	0.30	***	0.63	***	0.48	***

The caption describes all significant situations of correlation between parameters, including significant correlation (*) and extremely significant correlation (** and ***). The results in Table 3 showed that the parameters were highly correlated (***). ***, ** and * represent p< 0.001, p< 0.01and p< 0.05, respectively.

### 3.3 Changes in leaf nitrogen allocation

**Figure 3:**
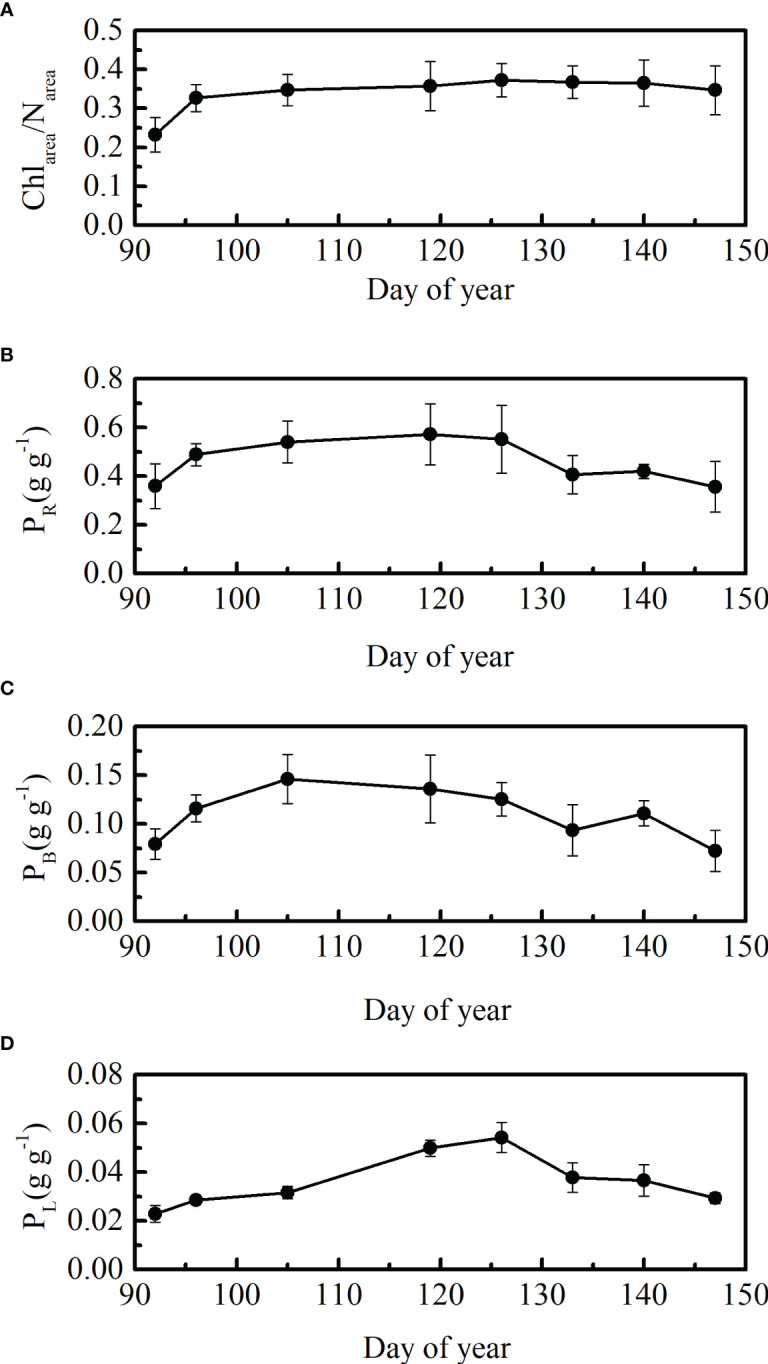
Seasonal patterns in **(A)** leaf Chl_area_ to N_area_ ratios, **(B)** P_R_, **(C)** P_B_, and **(D)** P_L_, ± SD, in 2021.

The ratios between leaf Chl_area_ and N_area_ indicate the allocation of leaf nitrogen between the Rubisco and leaf chlorophyll components (Kenzo et al., 2006). There was little seasonal variation in the ratios between leaf Chl_area_ to N_area_ (both units are μg cm^-2^) after DOY 105 in 2021 (**Figure 3A**). The leaf Chl_area_/N_area_ ratios showed a rapidly increasing trend at the beginning stage (DOY 92 and DOY 96). The ratios were 0.23 and 0.32 at DOY 92 and 96, respectively. Leaf Chl_area_/N_area_ ratios were maintained at approximately 0.36 from DOY 105 to DOY 147 (**Figure 3A**).

P_R_, P_B,_ and P_L_ showed seasonal patterns that first increased and then decreased (**Figures 3B–D**). The growing stage at which leaf P_B_ reached its highest point in winter wheat differed from that of P_R_ and P_L_. Temporal variations in leaf P_R_ and P_L_ were coordinated, reaching their highest points at the flowering stage. In general, changes in leaf N allocation to different N pools were dynamic (**Figures 3B–D**), which may have led to a weak correlation between leaf nitrogen and V_cmax25_ (**Table 3**).

### 3.4 Relationships among leaf nitrogen, chlorophyll and carotenoid contents

**Figure 4:**
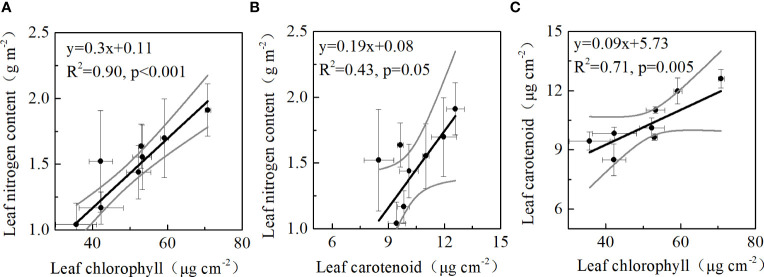
Relationships between **(A)** leaf nitrogen and chlorophyll contents, **(B)** leaf nitrogen and carotenoid contents, **(C)** leaf carotenoid and chlorophyll contents in 2021. Horizontal error bars denote standard deviation of leaf chlorophyll and leaf carotenoid. Vertical error bars refer to standard deviation of leaf nitrogen and leaf carotenoid.

A significant linear relationship between leaf nitrogen and leaf chlorophyll contents (R^2 ^= 0.90, p<0.001) was observed in our study. The observations on DOYs 92 and 96 were outside the 95% confidence intervals of the regression (**Figure 4A**), which may be attributed to the significant variations in nitrogen allocation to the leaf chlorophyll fractions on these two days (**Figure 3A**). Leaf Chl_area_ was also strongly correlated with Car_area_ (R^2 ^= 0.71, p=0.005) (**Figure 4B**). However, a weaker relationship between leaf N_area_ and Car_area_ was observed (R^2 ^= 0.43, p=0.05) in winter wheat in 2021 (**Figure 4C**).

### 3.5 The importance of each prediction variable to V_cmax25_

**Figure 5:**
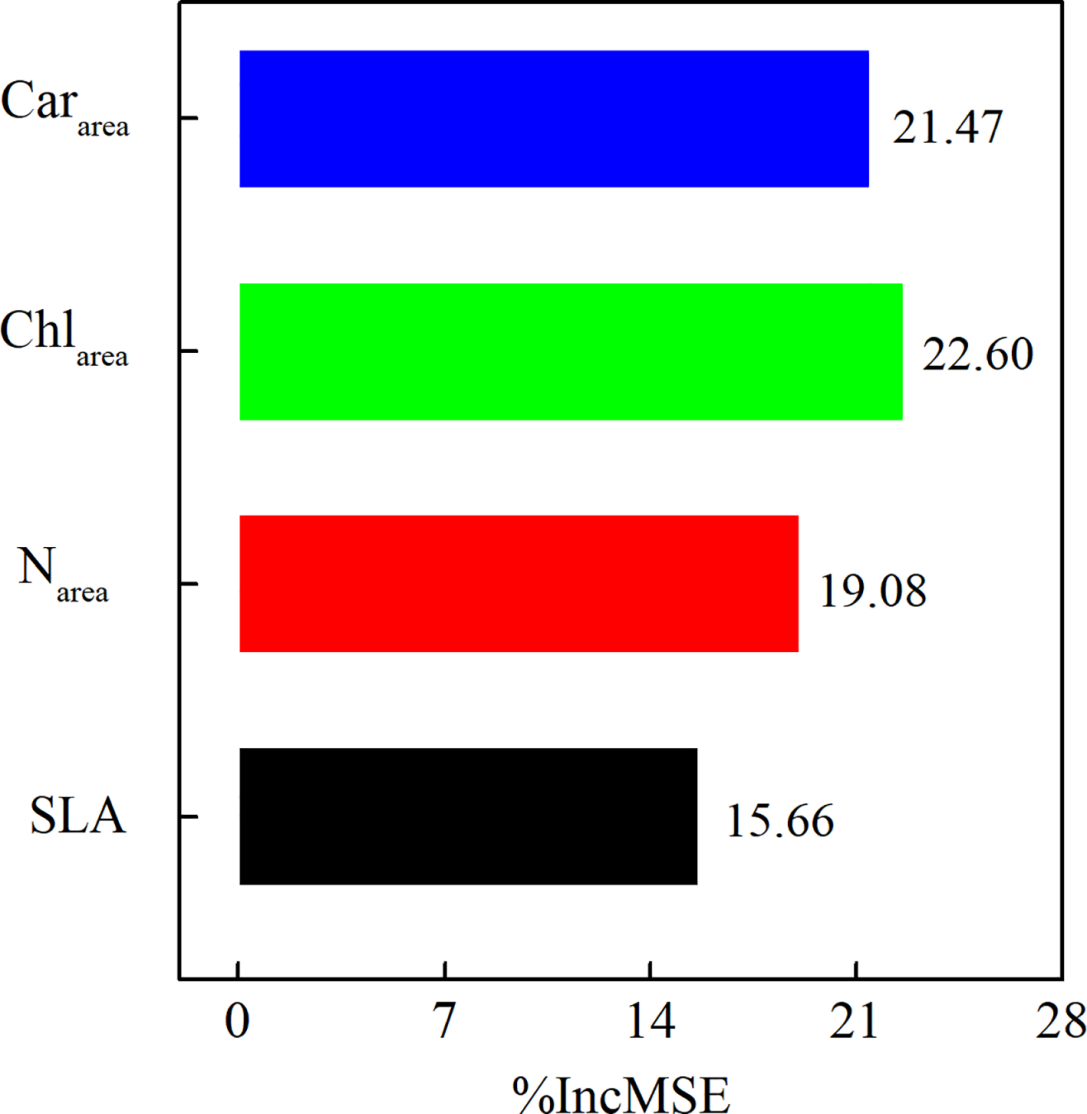
The importance of leaf Car_area_, leaf Chl_area_, leaf N_area_, and SLA to V_cmax25_ in 2021.

We used a random forest regression analysis to evaluate the relative importance of each prediction variable for V_cmax25_. Leaf Car_area_, Chl_area,_ and leaf N_area_ were all main prediction variables for V_cmax25_ in our study (**Figure 5**). Leaf Chl_area_ (%IncMSE = 22.60%) was the most important driver of V_cmax25_, followed by leaf Car_area_ (%IncMSE was 21.47%), and leaf N_area_ (%IncMSE = 19.08%). The importance of SLA (%IncMSE = 15.66%) to V_cmax25_ was far below the importance of leaf photosynthetic pigment and nitrogen content (**Figure 5**).

### 3.6 Optimization of V_cmax25_ model by multiple regression models

Multiple linear regression models were established to improve the accuracy of the V_cmax25_ models using leaf N_area_, Chl_area,_ and Car_area_ (Equations 6-9). The estimation accuracies of the binary linear regression models for V_cmax25_ (R^2 ^= 0.72, 0.70, and 0.69, respectively, for f(N_area_, Chl_area_), f(Chl_area_, Car_area_), and f(N_area_, Car_area)_) were all significantly higher than those of the two simple linear regression models for f(N_area_) and f(Car_area)_ (R^2 ^= 0.36 and 0.47, respectively) in our study (**Tables 3**, **4**). However, the estimation accuracy of the simple linear regression models for f(Chl_area_) (R^2 ^= 0.69) was not significantly different from that of the binary linear regression models (**Tables 3**, **4**). The model based on leaf N_area_, Chl_area,_ and Car_area_ had the highest accuracy in estimating V_cmax25_ (R^2 ^= 0.75, p<0.001), which was only 0.06 higher than that of the simple linear regression models for f(Chl_area_) (R^2 = ^0.69) (**Tables 3**, **4**). Thus, leaf Chl_area_ was a better predictor for V_cmax25_ than leaf N_area_ in winter wheat at the YC site (**Tables 3**, **4**). Incorporating leaf photosynthetic pigments (chlorophyll and carotenoid content) into photosynthetic models can significantly improve the estimation accuracy of V_cmax25_ model based on leaf nitrogen for winter wheat.

**Table 4:** Coefficient of determination (r^2^) of V_cmax25_ models based on different leaf traits variables.

V_cmax25_ models	R^2^	p-value
f(N_area_, Chl_area_)	0.72	<0.001
f(Chl_area_, Car_area_)	0.70	<0.001
f(N_area_, Car_area)_	0.69	<0.001
f(N_area_, Chl_area_, Car_area_)	0.75	<0.001


(6) $${\text{V}}_{\text{cmax}25}=15.17N_{\text{area}}+1.99{\text{Chl}}_{\text{area}}-34.43$$

(7) $${\text{V}}_{\text{cmax}25}=1.99{\text{Chl}}_{\text{area}}+3.13{\text{Car}}_{\text{area}}-43.51$$

(8) $${\text{V}}_{\text{cmax}25}=37.48{\text{N}}_{\text{area}}+12.68{\text{Car}}_{\text{area}}-98.92$$

(9) $${\text{V}}_{\text{cmax}25}=22.30{\text{N}}_{\text{area}}+1.26{\text{Chl}}_{\text{area}}+6.18{\text{Car}}_{\text{area}}-71.99$$

## 4 Discussion

### 4.1 Differences in seasonal trends of photosynthetic parameters

In our study, leaf A_sat_ and photosynthetic parameters appeared to follow trends in leaf chlorophyll content. However, there were some differences in the seasonal patterns of leaf chlorophyll, nitrogen, and carotenoid contents. Leaf N_area_ was relatively high at the elongation stage relative to leaf Chl_area_ (**Figures 1A, B**), which may be attributed to the inorganic nitrogen present in buds before leaf flushing. The different trends in leaf nitrogen and leaf chlorophyll maybe attributed to dynamic changes in leaf nitrogen partitioning among photosynthetic pools (Croft et al., 2017; Liu et al., 2023a). Fertilization management can also maintain high leaf nitrogen content at the start of season (Lu et al., 2020). Leaf Car_area_ showed smaller seasonal variations compared to leaf Chl_area_ in winter wheat, particularly after the flowering stage (**Figure 1B**). As there is massive loss of leaf chlorophyll in winter wheat during senescence in the filling stage, carotenoids are retained in the leaves for a much longer time (Wong et al., 2019). In the early growth stage, the leaf Chl_area_ is higher than the leaf Car_area_. The leaves appear green because green light is almost completely reflected. In the late stage, leaf chlorophyll is heavily damaged, but leaf carotenoids are only slightly affected, causing the leaves to turn yellow (Stylinski et al., 2002; Garrity et al., 2011). Flowering stage is an important physiological stage for winter wheat, since all the photosynthetic parameters have inflection point in this period. The results are consistent with Lu et al. (2020). The temperature rises gradually after the greening period. At this period, plant root is vigorous, enzyme activity and plant photosynthetic capacity increases. Thus, leaf photosynthetic parameters increase gradually and reach the maximum point at the flowering stage. The reproductive growth of winter wheat is dominant after flowering stage. Leaf and other vegetative organs gradually stop growing and aging. Therefore, leaf photosynthetic parameters decrease gradually (Yang et al., 2020).

### 4.2 Relationships among leaf V_cmax25_, nitrogen and photosynthetic pigments

Leaf nitrogen was closely correlated with leaf chlorophyll in previous studies, with a fixed value of leaf Chl_area_/N_area_ ratio (Sage et al., 1987; Evans, 1989; Houborg et al., 2013). Our results also showed a strong linear relationship between leaf Chl_area_ and N_area_ (R^2 ^= 0.90; p< 0.001) (**Figure 3A**). The robustness of the linear correlation between leaf nitrogen and chlorophyll contents was influenced by changes in leaf nitrogen allocation to chlorophyll (Lu et al., 2020). A relatively stable allocation of leaf nitrogen to leaf chlorophyll (Chl_area_/N_area_ ratio of approximately 0.36) was found in our study for winter wheat (**Figure 4A**), which contributed to a good linear relationship between leaf Chl_area_ and N_area_ (**Figure 3A**).

V_cmax25_ was closely related to leaf N_area_, leaf Chl_area_, and SLA in previous studies (Houborg et al., 2013; Houborg et al., 2015; Croft et al., 2017; Watanabe et al., 2018; Miner and Bauerle, 2019; Qian et al., 2021; Lu et al., 2022). However, the results are inconsistent in different studies, indicating that these relationships vary among species and are difficult to apply at large scales. Qian et al. (2021) showed a stronger linear relationship between V_cmax25_ and Chl_area_ than between leaf V_cmax25_ and N_area_ across 13 species. In other ecosystems, a strong relationship exists between leaf V_cmax25_ and N_area_ and the slopes vary among species (Walker et al., 2014; Quebbeman and Ramirez, 2016). However, the slopes of the relationship in N_area_‐V_cmax25_ varies greatly with environmental conditions and PFTs (Walker et al., 2014; Rogers et al., 2017). A weaker correlation between leaf V_cmax25_ and leaf N_area_ than between leaf V_cmax25_ and Chl_area_ were showed in our study (**Figure 2**; **Table 3**), which is in agreement with Qian et al. (2021). Rubisco-N allocation (P_R_), rather than the total leaf nitrogen content, was more related to V_cmax_ based on the meta-analysis (Ali et al., 2015). P_R_ showed significant seasonal variation during the growing season in our study (**Figure 4B**). The weak correlation between leaf N_area_ and V_cmax25_ may also be attributed to variations in P_R_ (**Figures 2**, **4B**; **Table 3**). Therefore, leaf N_area_ is not an ideal predictor for V_cmax25_ in the present study. The temporal variations in leaf P_L_ coordinated with changes in P_R_, which indicated that the allocation of leaf nitrogen to leaf carotenoids was dynamic. Consequently, A weak correlation between the leaf N_area_ and leaf Car_area_ for winter wheat area was showed in our study (**Figure 3B**).

### 4.3 Physiological mechanism for the relationships between leaf photosynthetic pigments and V_cmax25_

Our results showed a stronger correlation between V_cmax25_ with both leaf Chl_area_ (R^2 ^= 0.69) and Car_area_ (R^2 ^= 0.47) than with leaf N_area_ (R^2 ^= 0.36) (**Figure 2**; **Table 3**). Leaf photosynthetic pigments are a better predictor for V_cmax25_ in winter wheat. The underlying mechanism of this phenomenon is the driving role of leaf pigment in light harvesting of photosynthesis (Zhang et al., 2009; Gitelson et al., 2014; Li et al., 2018b). The random forest regression analysis also showed leaf chlorophyll and carotenoid contents were more important than leaf nitrogen content for V_cmax25_ (**Figure 5**). Compared with other leaf traits, V_cmax25_ can be accurately retrieved based on leaf chlorophyll content from remote sensing data (Gitelson et al., 2006; Croft et al., 2013). Moreover, leaf chlorophyll can effectively eliminate the influence of nonphotosynthetic nitrogen since it only reflects the changes of photosynthetic active N pool (Alton, 2017; Croft et al., 2017). Carotenoid, which is important component of plant photosynthesis, participate in the collection of sunlight, especially at wavelengths where leaf chlorophyll molecules are not absorbed strongly (Ritz et al., 2000). Leaf carotenoid also protect chlorophyll molecules from photo-oxidation. Carotenoid is commonly referred to as “auxiliary pigments” in light harvesting center, promoting the transfer of excitation energy to the reaction center (Niyogi et al., 1997). Leaf chlorophyll and carotenoid molecules are usually arranged in clusters to maximize the capture of light energy (Croft and Chen, 2018).

Leaf chlorophyll and carotenoid contents are the most important factors determining photosynthetic rate, owing to their important roles in light capture and absorption of photosynthetic effective radiation (Zhang et al., 2009; Zhang et al., 2011; Kooistra and Clevers, 2016). Therefore, leaf photosynthetic pigments play an important role in simulating vegetation productivity processes (Croft et al., 2017; Luo et al., 2018). The construction of a V_cmax25_ model based on photosynthetic pigments can improve the accuracy of ecological process model simulations (Luo et al., 2019; Liu et al., 2023b). The multiple linear regression models established in our study showed that f((N_area_, Chl_area_, Car_area_) had the highest optimization accuracy for the V_cmax25_ model (R^2 ^= 0.75), which represents different information expressed by the leaf N_area_ and leaf photosynthetic pigment. The estimation accuracy of the V_cmax25_ model based on N_area_, Chl_area_, and Car_area_ (R^2 ^= 0.75) was only 0.05 higher than that of the V_cmax25_ model based on Chl_area_ and Car_area_ (R^2 ^= 0.70). However, the estimation accuracy of the V_cmax25_ model based on Chl_area_ and Car_area_ (R^2 ^= 0.70) was 0.34 higher than that of the V_cmax25_ model based on N_area_ (R^2 ^= 0.36). Leaf photosynthetic pigments can significantly improve the estimation accuracy of V_cmax25_ based on leaf nitrogen in winter wheat.

## Data availability statement

The raw data supporting the conclusions of this article will be made available by the authors, without undue reservation.

## Author contributions

YL analysed date and wrote the manuscript. The experiments were designed by TQ. QW and TF performed the experiments. YQ and LH revised the manuscript. All authors contributed to the article and approved the submitted version.
